# Kinetic modeling of *Shewanella baltica* KB30 growth on different substrates through respirometry

**DOI:** 10.1186/s12934-017-0805-7

**Published:** 2017-11-03

**Authors:** Juan Carlos Leyva-Díaz, José Manuel Poyatos, Paolo Barghini, Susanna Gorrasi, Massimiliano Fenice

**Affiliations:** 1 Department of Civil Engineering, University of Granada, 18071 Granada, Spain; 2 Institute for Water Research, University of Granada, 18071 Granada, Spain; 3 Department of Ecological and Biological Sciences, University of Tuscia, Largo Università snc, 01100 Viterbo, Italy; 4 Laboratory of Applied Marine Microbiology, ConISMa, University of Tuscia, 01100 Viterbo, Italy

**Keywords:** Carbon utilization, Kinetics, Modeling, *Shewanella baltica*

## Abstract

**Background:**

*Shewanella baltica* KB30 was isolated from seawater collected in Kandalaksha Bay, White Sea (Russia). This strain is known for its ability to grow on a pool of different substrates, including carbohydrates, carboxylic and amino acids, and lipids. However, no data are available on its metabolic efficiency in relation to the use of different carbon sources typologies. This work represents the first attempt to characterize *S. baltica* by its heterotrophic kinetic performance.

**Results:**

Growth and substrate consumption, during the biodegradation of sodium acetate, glucose, tween 80 and peptone, were analyzed through a respirometric method. To find the model best fitting the experimental data and to obtain the kinetic parameters, the equations of Monod, Moser, Contois and Tessier were applied. The kinetic behavior of *S. baltica* was fitted to Monod model for sodium acetate and tween 80, while it was adjusted to Contois model for glucose and peptone. In this regard, peptone was consumed faster than the other substrates, as indicated by the highest values of substrate degradation rate, which exceeded 60 mg O_2_ L^−1^ h^−1^.

**Conclusions:**

Proteolytic metabolism was favored than lipidic and glucidic metabolism, which could contribute much more to mineralization and recycling of proteins than lipids and carbohydrates.

## Background

*Shewanella* was proposed as a new genus in 1985 and it was named in honor of James Shewan, as acknowledgment of his contributions to fisheries microbiology [1, 2]. This genus belongs to the family Shewanellaceae, order Alteromonadales, within the γ-Proteobacteria class [3, 4]. These bacteria are motile Gram-negative rods and H_2_S-producers; in general, they are non-fermenting bacteria, although glucose fermentation has been reported [5, 6].

Mesophilic, psychrotolerant, psychrophilic or barophilic *Shewanella* species have been isolated from various habitats [7, 8]. One of the most representative psychrotolerant (psychrotrophic) species is *Shewanella baltica* [9], which is widespread in marine environments and has been isolated from some fresh and ice-stored fish [10, 11]. *Shewanella* species, in particular *S. baltica*, together with other specific spoilage organisms (i.e. *Pseudomonas* spp.), are involved in the spoilage of seafood products causing offensive off-flavors and the production of toxic compounds [12–14]. *S. baltica* is the most represented species among H_2_S-producing organisms in stored marine fish and has the capacity to produce signal compounds to facilitate the potential spoilage activity [7, 14].

Many works dealt with the metabolic competences of Shewanella, some studies extensively investigated its metabolism using both phenotypic methods (i.e. Biolog Microarray and API) and—omic sciences; substrate utilization by this genus and the related pathways had been mapped [15] together with various complete genomes [16] supplying a detailed insight of its ecophysiology and adaptive evolution. Phenotypic tests permit to verify the congruence between predicted and observed phenotype, and to understand the actual ability to utilize a single substrate.

However, one of the basic tools in microbiology is the study of the relationships between the use of different substrates and the relative specific growth rate, giving information on the efficiency of substrate consumption in relation to different metabolic strategies. This kind of investigation, possibly supported by adequate kinetic modeling, is helpful to understand the microbial approach in its primary metabolism and to predict possible biodegradation of organic compounds in natural and engineered environments [17].

In this context, a very useful tool is respirometry which allows indirect assessment of the aerobic metabolism of a given substrate by monitoring the biological oxygen consumption under well-defined conditions. Estimation of the kinetic parameters, through exogenous oxygen uptake rate curves, is also obtained. This method consists of measuring the dissolved oxygen (DO) concentration after the addition of determined amounts of substrate into the system [18, 19]. In other words, respirometry measures the “speed” of substrate consumption; comparison of respirometric experiments, carried out on different substrates, supplies information somehow regarding the “preferred and or favored” microorganism’s metabolism. This information is not given by the techniques mentioned above.

It is worth noting that, for *Shewanella* strains, there is a lack of predictive kinetic studies: only a few works modeled their kinetic performance concerning different substrate consumption [20, 21]. Moreover, to the best of our knowledge, no study based on respirometric methods is available for *S. baltica*.

*Shewanella baltica* KB30, isolated from seawater collected at the Arctic Circle (Kandalaksha Bay, White Sea, Russia) has been previously investigated for some physiological and metabolic competences [8, 22]. The strain showed an uncommon adaptation to temperature variations, growing from 0 to over 35 °C, but above all, revealing optimal or sub-optimal growth in a very wide range of temperatures (15–30 °C), reflecting adaptation to the environmental peculiarities of that sub-extreme region [8]. In view of potential applications, together with those of other bacteria from same area, its substrate utilization pattern was profiled using the Biolog Microarray System [22].

The aim of the present study was to provide a more clear understanding of the primary metabolic strategies of this new cold-adapted strain of *S. baltica* on selected substrates. Thus, in this work, the aerobic heterotrophic kinetics of strain KB30 was studied by respirometry using different models (Monod, Moser, Contois and Tessier) to compare the consumption rate of various organic substrates and to predict bacterial biomass production. Respirometric experiments were carried out in a stirred tank bioreactor under controlled batch conditions.

## Methods

### Microorganism and culture conditions

The strain of *S. baltica* KB30 used in this study was isolated in a previous work from seawater collected in Kandalaksha Bay (White Sea, Russia) [8], maintained at 4 °C in the microorganism culture collection of DEB (Department of Ecological and Biological Sciences, University of Tuscia) and sub-cultured on plate count agar (PCA, Difco, USA) slants when necessary.

### Bioreactor inoculum preparation

Biomass for liquid cultures (pre-inoculum) was obtained in PCA plates (incubated at 20 °C for 24 h), from PCA slants, by the streak method. After incubation, biomass was recovered by sterile spatulas and transferred into 250 mL Erlenmeyer flasks filled with 50 mL of M9 minimal saline medium supplemented with 10 g L^−1^ of glucose. Flasks were then incubated at 20 °C and 150 rpm in an orbital shaker (24 h) to obtain the adequate cell density (OD_600_ = 6.0). Biomass from flasks was then harvested by centrifugation (7000 rpm for 10 min), washed twice with M9 (without glucose), re-suspended in 10 mL of the same medium and added to the bioreactor to a cell density of ca. 0.3 (OD_600_). Cell density, used for both the flask and bioreactor cultures, was measured through spectrophotometry (OD_600_). The calibration curve was done with bacterial counts.

### Experimental system and respirometric assays

**Fig. 1:**
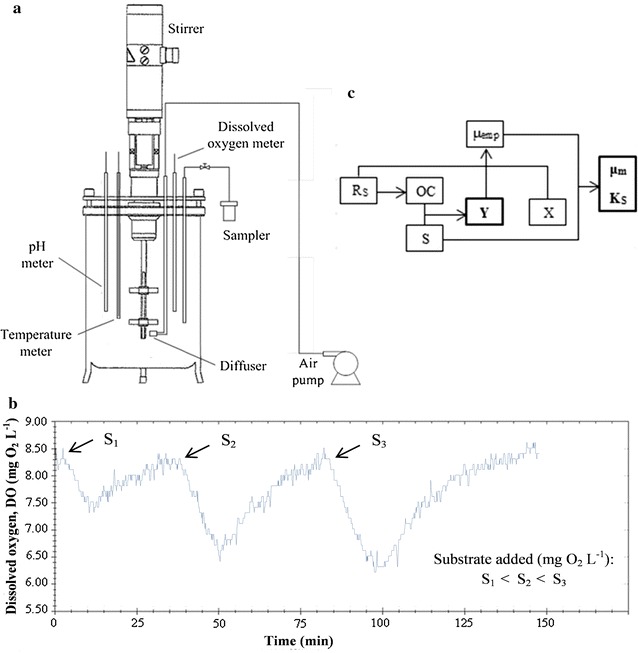
Schematic diagram of the bioreactor used in the respirometric experiments (**a**); evolution of dissolved oxygen (DO) (**b**) and schematic diagram of the assessment of the kinetic parameters for the biodegradation of different substrates by *Shewanella baltica* KB30: yield coefficient (Y), substrate concentration S, biomass concentration (X), oxygen consumption (OC), maximum specific growth rate (µ_m_), empirical specific growth rate (µ_emp_), dynamic oxygen uptake rate (R_s_) and substrate half-saturation coefficient (Ks) (**c**)

The bioreactor used for the respirometric experiments consisted of an Applikon 3 L (total volume) autoclavable stirred tank reactor controlled by an ADI 1010 Bio Controller and an ADI 1025 Bio Console (Applikon Dependable Instruments BV, NL). The bioreactor was equipped with autoclavable polarographic DO (Ingold, CH) and pH (Hamilton, USA) probes (Fig. 1a). The ADI 1010 Bio Controller was set up for automatic temperature (20.0 ± 0.1 °C) and stirrer speed control (300 rpm, six-bladed Rushton-type impellers). Aeration was maintained at 1.0 vvm by a rotameter. Since pH in the bioreactor was stable throughout the experiment (7.25 ± 0.50), pH control was not necessary.

One liter of M9 was prepared, transferred to the bioreactor, inoculated as reported above and aerated for 4 h to achieve a stable DO concentration.

Bioprocess parameters (pH, DO, agitation and temperature) were acquired using Biowatch software (Applikon Dependable Instruments BV, NL).

Respirograms show the consumption of DO as a result of bacterial respiratory metabolism in relation to a given carbon source. For the accurate characterization of bacterial heterotrophic kinetic behavior, in relation to the different nutrients, different concentrations of the same substrate are required. Due to the microbial metabolism, each addition of organic substrate should produce a DO decrease to a minimum value. Then, DO must increase again until a stable value is reached, which is achieved when the organic substrate is totally metabolized (Fig. 1b). The use of diverse substrates is important to evaluate different rates of respiratory metabolism [23, 24]. During respirometric experiments cultures were under limited but not limiting carbon source conditions.

Stock solutions of sodium acetate (5.0 g L^−1^), glucose (2.5 g L^−1^), tween 80 (0.2 g L^−1^) and peptone (1.0 g L^−1^) were prepared in order to establish the same gradient of substrate concentration (S). Since *S. baltica* KB30 can use for its growth a very differentiated pattern of carbon sources, including organic acids, sugars, lipids and amino acids [22], the substrates for respirometric investigations had been chosen as prototypic compounds of principal cell metabolism (glucidic, proteolytic and lipolytic). As required by the kinetic method, the concentration of the different organic substrates was expressed as the chemical oxygen demand (COD) (mg O_2_ L^−1^), determined in accordance with standard methods [25].

The respirometric tests for *S. baltica* KB30 were carried out using three different concentrations of each substrate (S_1_, S_2_ and S_3_) prepared by diluting the stock solutions (35, 70 and 100%, respectively). For the various substrates, 10 mL of each dilution were added in sequence to the inoculated M9 when DO reached a maximum steady level (Fig. 1b), sign of total substrate consumption by KB30. Corn steep liquor (0.1 mL) was added to each experiment to improve cell growth. The experiments were carried out in triplicate.

**Fig. 2:**
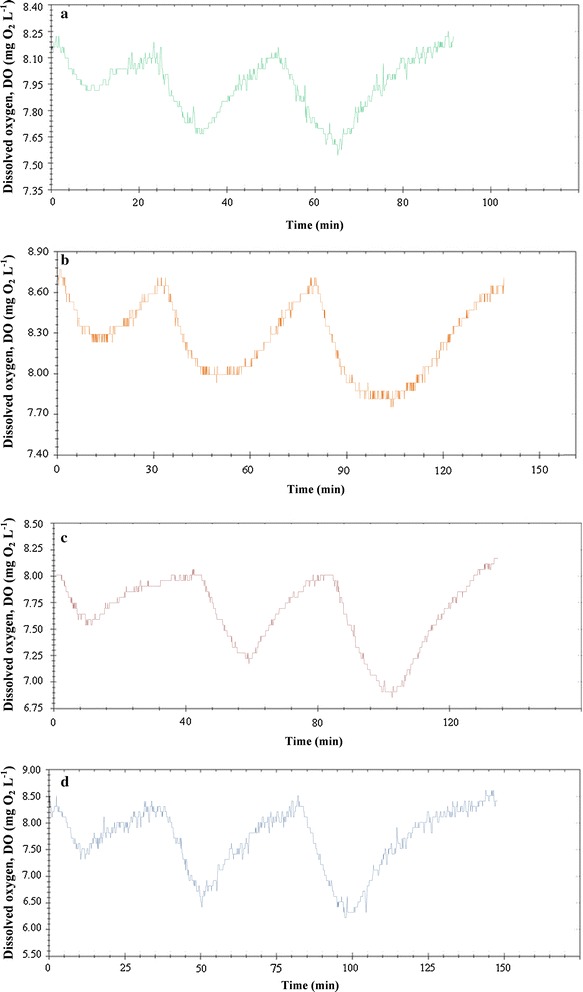
Time course of dissolved oxygen (DO) in a typical respirometric experiment carried out with *Shewanella baltica* KB30 for the metabolization of the selected substrates: sodium acetate (**a**), glucose (**b**), tween 80 (**c**) and peptone (**d**)

The time course of DO, measured as percentage saturation, was monitored and converted into respirograms (time course of DO concentration) corresponding to the different kinetic experiments related to sodium acetate, glucose, tween 80 and peptone (Fig. 2).

### Kinetic modeling

For a more comprehensive characterization of the heterotrophic kinetics, the models of Monod, Moser, Contois and Tessier were applied and compared. These models allowed adjustment of the process kinetics for the different substrates tested. In this regard, the purpose of using different models was to understand which one permitted a more efficient empirical interpretation of the biodegradation of the various substrates since each model is fitted to a different kinetic behavior. The best fitting model for the experimental data was chosen based on the lowest least-squared error (LSE) between empirical and theoretical data:

$$\sum\limits_{{{\text{i}} = 1}}^{\text{n}} {\left( {\upmu_{\text{theoretical}} - \upmu_{\text{emprical}} } \right)}^{2} .$$

In light of this, the weighted sum of squares of differences between the empirical and theoretical values was minimized to yield the most appropriate kinetic parameters for the considered model [26]. In order to compare the efficiency of the different models, the empirical specific growth rate (µ_empirical_) was calculated from the respirograms obtained for each substrate.

The respirometric experiments permitted the estimation of the following kinetic parameters: yield coefficient referred to the total suspended solids (TSS), Y_TSS_ (mg TSS mg COD^−1^), maximum specific growth rate, μ_m_ (h^−1^) and substrate half-saturation coefficient, K_S_ (mg O_2_ L^−1^).

The dynamic oxygen uptake rate (R_S_, mg O_2_ L^−1^ h^−1^) was obtained through the derivation of DO depending on the time for each respirogram corresponding to the different substrates. The oxygen consumption (OC, mg O_2_ L^−1^) was determined from the numerical integration of R_S_ for each addition (dilution) of organic substrate, as shown in Eq. (1):

1 $${\text{OC = }}\int\limits_{{{\text{t}}_{0} }}^{\text{t}} {{\text{R}}_{\text{s}} } \;{\text{dt}} .$$

According to Helle [23], the yield coefficient referred to oxygen, ${\text{Y}}_{{{\text{O}}_{2} }} ,$ is calculated as indicated in Eq. (2):

2 $${\text{Y}}_{{{\text{O}}_{2} }} = \frac{{{\text{S}} - {\text{OC}}}}{\text{S}}.$$

The value of Y_TSS_ was obtained through Eq. (3):

3 $${\text{Y}}_{\text{TSS}} = \frac{{{\text{Y}}_{{{\text{O}}_{2} }} }}{{{\text{f}}_{\text{cv}} }}$$

where f_cv_ is a conversion factor (1.48 mg COD mg TSS^−1^).

The value of µ_empirical_ was determined considering the relation between the cell growth rate (r_x_, mg TSS L^−1^ h^−1^) and substrate degradation rate (r_su_, mg O_2_ L^−1^ h^−1^), according to Leyva-Díaz et al. [24]. The calculation of r_su_ was carried out from the derivation of S depending on the time in Eq. (2). The value of µ_empirical_ is shown in Eq. (4):

4 $$\upmu_{\text{emprical}} = \frac{{{\text{Y}}_{\text{TSS}} \cdot {\text{R}}_{\text{s}} }}{{\left( {1 - {\text{Y}}_{{{\text{O}}_{2} }} } \right) \cdot {\text{X}}_{\text{T}} }}$$

where X_T_ is the biomass concentration (mg TSS L^−1^).

Biomass concentration was calculated from the OD_600_ of samples (2 mL) taken before each addition of substrate. A calibration curve, correlating biomass concentration (as total suspended solids, TSS) and OD_600_, was prepared using several dilutions of *S. baltica* KB30 biomass. The biomass concentration (TSS) of the various dilutions was analyzed according to the APHA [25].

Equation (5) shows the relation between biomass concentration and OD_600_ for *S. baltica* KB30:

5 $${\text{X}}_{\text{T}} \left( {{\text{mg}}\;{\text{TSS L}}^{ - 1} } \right) = 2140.3 \cdot {\text{OD}}_{600} - 325.2$$

The correlation coefficient (R^2^) between X_T_ and OD_600_ was 0.9995.

The concentrations of the different substrates, expressed as COD, and the biomass concentrations, measured as TSS, are indicated in Table 1.

**Table 1:** Values of chemical oxygen demand (COD), substrate (S) and biomass (X_T_) concentrations for the various dilutions (S1, S2 and S3) of substrates used in the kinetic study

Substrate	Dilution	COD (mg O_2_ L^−1^)	S (mg O_2_ L^−1^)	X_T_ (mg TSS L^−1^)
Sodium acetate	S1	630	6.1765	306.2
	S2	1260	12.2330	323.3
	S3	1800	17.3077	443.1
Glucose	S1	1190	11.6667	351.1
	S2	2380	23.1068	374.7
	S3	3400	32.6923	500.9
Tween 80	S1	385	3.7745	146.6
	S2	770	7.4757	178.7
	S3	1100	10.5769	239.8
Peptone	S1	840	8.2353	175.6
	S2	1680	16.3107	265.5
	S3	2400	23.0769	284.8

The kinetic parameters μ_m_ and K_S_ were assessed according to the schematic diagram shown in Fig. 1c, as indicated by Leyva-Díaz et al. [24].

The theoretical specific growth rate (µ_theoretical_) was evaluated for each model by using the Solver add-in facility of Microsoft Office Excel, by considering Eqs. (6)–(9).

The Monod model empirically establishes a saturation-type equation to describe the specific rate of microbial growth in relation to the concentration of a limiting substrate [20], according to Eq. (6):

6 $$\upmu = \upmu_{\text{m}} \frac{\text{S}}{{{\text{K}}_{\text{S}} + {\text{S}}}}$$

where μ is the specific growth rate (h^−1^).

A different approach for assessing the kinetic parameters is the Moser model, which is also an unstructured kinetic model (as is the Monod model) and is also based on S [20]. This model is described by Eq. (7):

7 $$\upmu = \upmu_{\text{m}} \frac{{{\text{S}}^{\text{n}} }}{{{\text{K}}_{\text{S}} + {\text{S}}^{\text{n}} }}$$

where *n* is a constant as the exponent of S. The Moser model was studied for n = 2.

The Contois model describes an inverse relation between the microbial concentration and its specific growth rate, described by Eq. (8) [27], as the specific growth rate decreases when the microbial concentration increases:

8 $$\upmu = \upmu_{\text{m}} \frac{\text{S}}{{{\text{K}}_{\text{C}} {\text{X}}_{\text{T}} + {\text{S}}}}$$

where K_C_ is a growth coefficient of the Contois function.

The value of μ depends on the substrate and biomass concentrations. However, μ is only a function of S in the Monod model.

The Tessier model is another unstructured model, which relates µ and S through an exponential function [28], according to Eq. (9):

9 $$\upmu = \upmu_{\text{m}} \left( {1 - {\text{e}}^{{ - \frac{\text{S}}{{{\text{K}}_{\text{s}} }}}} } \right)$$

The value of r_su_ was obtained from the relation between the biomass produced and the substrate consumed indicated through Y_TSS_, according to Eq. (10):

10 $${\text{r}}_{\text{su}} = - \frac{{\upmu \cdot {\text{X}}_{\text{T}} }}{{{\text{Y}}_{\text{TSS}} }}$$

### Statistical analysis

When necessary, data were analyzed with a one-way analysis of variance (ANOVA) and significance of differences was assessed by pair-wise multiple comparison procedure (Tukey test). Statistical analysis was carried out by the software SigmaStat 2.0 (Jandel, San Rafael, CA, USA).

## Results and discussion

**Fig. 3:**
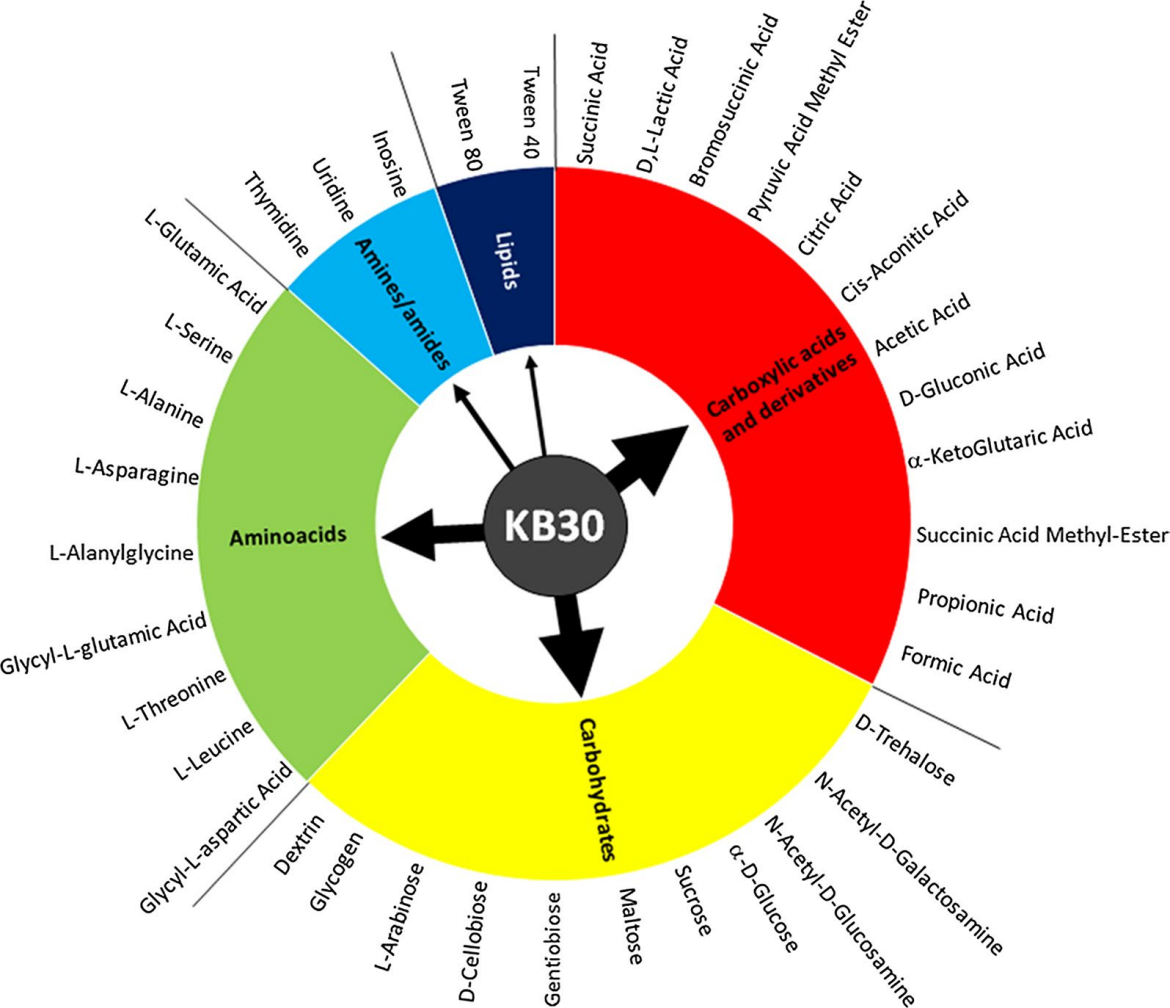
Degrading ability of *Shewanella baltica* KB30 regarding different substrate categories (carbohydrates, carboxylic and amino acids, amine/amides and lipids), as previously determined by the Biolog Microarray system. Arrows’ thickness is proportional to the number of substrates used for each category

The ability of strain KB30 to use a wide array of substrates (95) had been tested by Pesciaroli et al. [22] using the Biolog Microarray System together with those of many other Arctic bacteria. However, the KB30 specific metabolic competences had never been discussed in details and no further investigations, indicating which metabolic choices are favored by the strain, were done. *S. baltica* KB30 was able to degrade a number (37) of carbon sources (Fig. 3) in line with the outcome of Deng et al. [29]. These authors, in the frame of the “Shewanella Federation” consortium, performed an extensive study, carrying out a detailed genetic and physiological characterization of various *S. baltica* strains. They stated that the following carbon sources were used by all studied strains: cis-aconitic acid, sucrose, d-gluconic acid, l-glutamic acid, dextrin, maltose, α-d-glucose, l-serine, *N*-acetyl-d-glucosamine, lactic acid, and inosine. Strain KB30 shared these features; most of them were observed also in other *S. baltica* strains studied by the Biolog Microarray System [9] or by extensive genomic/metabolic analyses [15]. The use of glycogen, gentiobiose, cellobiose, sucrose, d-gluconate, and citrate, included in the initial description of this species [9], was confirmed for KB30 also. On a numerical basis, the favorite carbon sources were carboxylic acids, carbohydrates and amino acids with 12, 11 and 9 compounds, respectively. However, the number of carbon source used for growth is not sufficient to represent the efficiency of the bacterial metabolism to degrade the different substrates and, consequently, which metabolic pathways could be considered as preferential. For these reasons, the KB30 heterotrophic metabolism was analyzed by respirometric/kinetic methods, using representative substrates of the mentioned different categories. The selection was also guided by possible potential application of the study.

Table 2 shows the kinetic parameters corresponding to the various models characterizing the biodegradation of the different substrates by *S. baltica* KB30. The accuracy of the models is given through the LSE values. By applying these models for the kinetics of cell growth and substrate consumption, a description of cellular behavior and substrate degradation was provided under different organic sources [21].

**Table 2:** Kinetic parameters according to the models of Monod, Moser, Contois and Tessier for the characterization of *Shewanella baltica* KB30 metabolization of the different substrates

Substrate	Model	µ_m_ (h^−1^)	K_S_ (mg O_2_ L^−1^)	Y_TSS_ (mg TSS mg OD^−1^)	LSE (h^−2^)
Sodium acetate	Monod	0.1163 ± 0.0029^a^	4.9267 ± 0.3518^a^	0.6695 ± 0.0295^a^	1.0661 × 10^−6^
	Moser	0.0946 ± 0.0024^a^	17.8571^(1)^ ± 1.2750^a^		9.4331 × 10^−6^
	Contois	0.1385 ± 0.0035^a^	0.0230^(2)^ ± 0.0016^a^		3.1754 × 10^−5^
	Tessier	0.0934 ± 0.0024^a^	5.3119 ± 0.3793^a^		6.1539 × 10^−6^
Glucose	Monod	0.0840 ± 0.0021^b^	1.6077 ± 0.1148^b^	0.6681 ± 0.0294^a^	2.5382 × 10^−5^
	Moser	0.0811 ± 0.0020^b^	14.3311^(1)^ ± 1.0232^a^		2.1119 × 10^−5^
	Contois	0.0886 ± 0.0022^b^	0.0071^(2)^ ± 0.0005^b^		1.7037 × 10^−5^
	Tessier	0.0799 ± 0.0020^b^	4.6965 ± 0.3353^a^		1.7588 × 10^−5^
Tween 80	Monod	0.1162 ± 0.0029^a^	0.5780 ± 0.0413^b^	0.6678 ± 0.0294^a^	6.4165 × 10^−5^
	Moser	0.1107 ± 0.0028^c^	1.3073^(1)^ ± 0.0933^b^		7.4084 × 10^−5^
	Contois	0.1202 ± 0.0030^c^	0.0046^(2)^ ± 0.0003^c^		8.1371 × 10^−5^
	Tessier	0.1089 ± 0.0027^c^	1.3941 ± 0.0995^b^		8.3179 × 10^−5^
Peptone	Monod	0.2815 ± 0.0071^c^	10.2421 ± 0.7313^c^	0.6687 ± 0.0294^a^	7.6023 × 10^−4^
	Moser	0.2032 ± 0.0051^d^	39.9392^(1)^ ± 2.8517^c^		1.0938 × 10^−3^
	Contois	0.2062 ± 0.0052^d^	0.0039^(2)^ ± 0.0003^c^		1.9104 × 10^−4^
	Tessier	0.2144 ± 0.0054^d^	9.6725 ± 0.6906^c^		9.0604 × 10^−4^

The kinetic parameters assessed were μ_m_ (maximum specific growth rate), K_S_ (substrate half-saturation coefficient) and Y_TSS_ (yield coefficient referred to total suspended solids), with the evaluation of LSE (least-squared error). Data are the mean of three replicates (± SD). For each kinetic model, column means followed by the same superscript letter were not significantly different (P > 0.01) as determined by the Tukey test

^(1)^K_S_ (mg O_2_ L^−1^)^2^ in the Moser model (for n = 2)

^(2)^K_C_ (mg O_2_ mg TSS^−1^) replaces K_S_ in the Contois model

Monod’s was the best fitting model to describe the kinetic performance of KB30 in relation to the biodegradation of sodium acetate and tween 80, as it showed the lowest LSE with values of 1.0661 × 10^−6^ h^−2^ and 6.4165 × 10^−5^ h^−2^, respectively (Table 2). For glucose and peptone, the best adjustment for the kinetic behavior was obtained by the Contois model, showing LSE values of 1.7037 × 10^−5^ h^−2^ and 1.9104 × 10^−4^ h^−2^, respectively (Table 2). LSE values are often used to determine the quality of model fitting of experimental data (concerning µ_empirical_), as reported by Annuar et al. for the kinetic modeling of bacteria such as *Pseudomonas putida* [28].

Very scarce information are available concerning the kinetic performance of *Shewanella* species in relation to the utilization of various substrates. However, even with the limited existing information, it is possible to underline consistent metabolic differences between strain KB30 and others of same genus. Both the Monod and Moser models were used by Miroliaei et al. [20] to characterize the performance of microbial fuel cells constituted by *Shewanella* sp. and *Escherichia coli,* describing their growth and substrate uptake kinetics in batch experiments. The values of µ_m_, obtained by these authors for *Shewanella* sp. regarding glucose metabolization (1.54 and 0.72 h^−1^ for the Monod and Moser models, respectively), were higher than those obtained in this study for *S. baltica* (0.0840 ± 0.0021 and 0.0811 ± 0.0020 h^−1^ for the Monod and Moser models, respectively), as indicated in Table 2. This difference could be related either to different operational and experimental conditions, affecting the bacterial dynamics and metabolism, or to actual diverse metabolic competences between the two strains.

Tang et al. studied *Shewanella oneidensis* MR-1 in minimal media by biochemical methods [21]. With acetate, they achieved a µ_m_ value (0.28 h^−1^) higher than those obtained in this study, which ranged from 0.0934 ± 0.0024 to 0.1385 ± 0.0035 h^−1^ (Table 2). Thus, *S. oneidensis* MR-1 metabolizes acetate faster than *S. baltica* KB30.

**Fig. 4:**
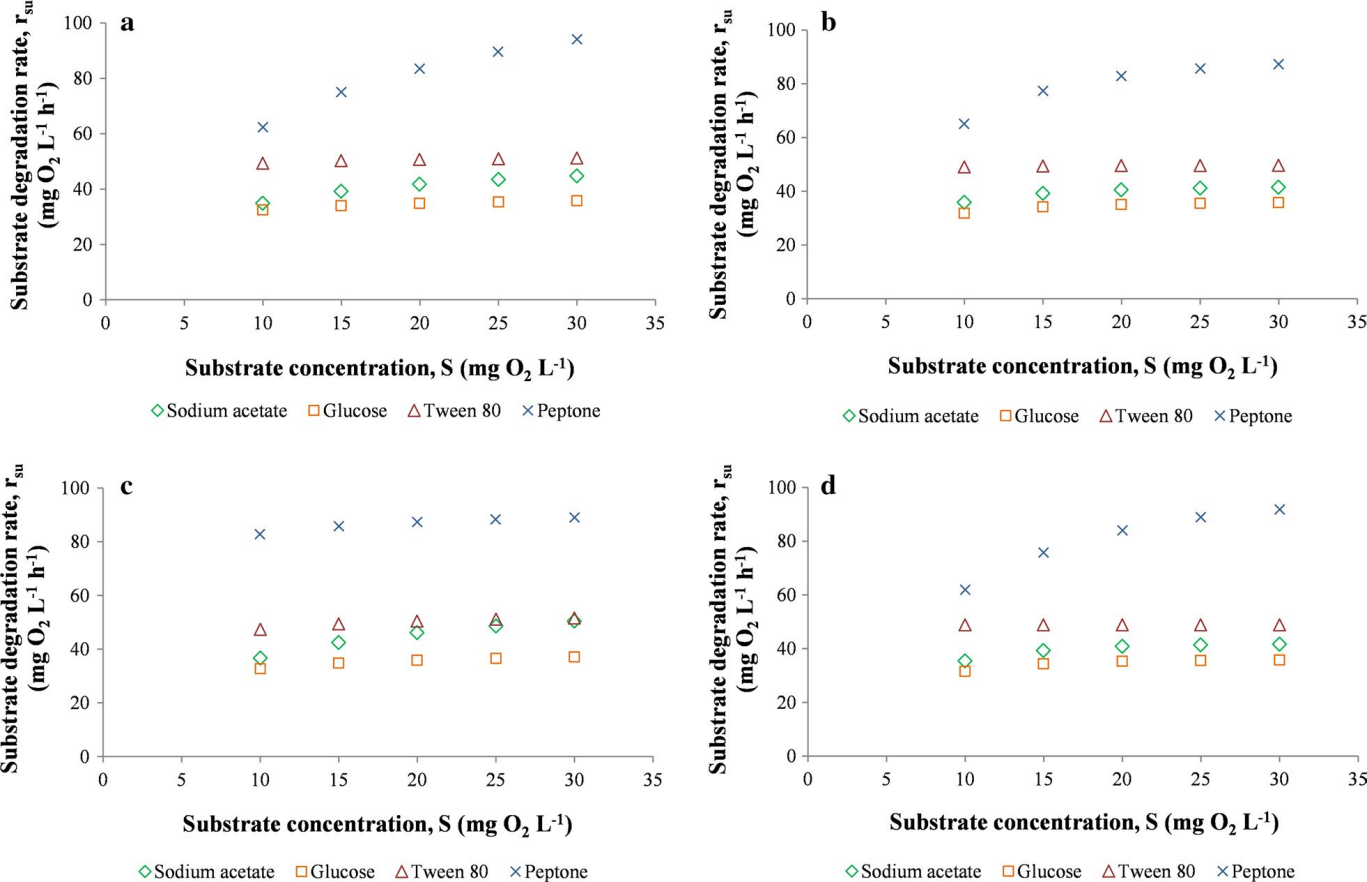
Substrate degradation rate (r_su_) of *Shewanella baltica* KB30 during the respirometric experiments in relation to substrate concentration (S), measured as chemical oxygen demand in (mg O_2_ L^−1^), obtained by different models: Monod (**a**), Moser (**b**), Contois (**c**) and Tessier (**d**)

Considering the different kinetic models, consumption of each substrate was analyzed through r_su_, as observed in Fig. 4. The bacterium clearly degraded the various organic compounds at different rates: for all the kinetic models analyzed, r_su_ was highest with peptone, followed by tween 80, sodium acetate and glucose (Fig. 4). µ_m_ is the maximum specific growth rate on a given substrate. Its lowest value for glucose indicated that this compound was biodegraded more slowly than the other substrates, as r_su_ is directly proportional to µ_m_, according to Eqs. (6)–(10). Therefore, the assimilation of peptone was faster than that of the other carbon sources. Peptone is an organic substrate (protein hydrolysate) containing nitrogen sources such as free amino acids and oligopeptides. Therefore, in strain KB30 proteolytic metabolism was probably faster than lipolytic and glucidic metabolism, as confirmed by the respirometry results related to tween 80 and glucose (Fig. 4). Bacteria with very high proteolytic capacity, accompanied by extremely scarce ability to degrade sugars and related compounds, are known, as shown by Juárez-Jiménez et al. for *Delftia tsuruhatensis* BM90, which is practically unable to degrade carbohydrates [30]. Strain KB30 is known for its ability to use a complex pattern of carbon sources including various sugars, amino acids, lipids and organic acids, showing a marked composite metabolism [22], but the respirometry results suggest that its proteolytic pathways could be favored. On the other hand, the faster metabolism on peptone could be justified by the higher bioavailability of its nitrogen sources, if compared with that contained in M9 (NH_4_Cl).

The rather good capacity of strain KB30 to metabolize lipids, even with a lower rate than that of peptone (Fig. 4), could find exploitation in biotechnology. An interesting application for environmental issues would be the biological removal of pollutant compounds chemically correlated to tween 80 (C_64_H_124_O_26_, PM 1310 g mol^−1^), such as surfactants and/or emulsifiers. Since KB30 is able to use a wide pattern of lipids and/or fatty acids [22], this applicative potential would be even higher.

The pattern obtained by the kinetic study indicated that carbohydrate metabolism of strain KB30 was rather slow, as shown by the lowest value of r_su_ for glucose (Fig. 4).

The kinetic results obtained in this work somehow reflect those stated by Pesciaroli concerning the strain KB30 extracellular enzyme pattern [31]. The bacterium was able to produce proteases and lipases, while no production of hydrolytic enzymes targeting natural polysaccharides (such as cellulose, starch, pectin and chitin) was detected. This probably suggests that its role in nature is more oriented to the mineralization and recycling of proteic and lipidic matrices than to carbohydrates and glucidic substrates.

**Fig. 5:**
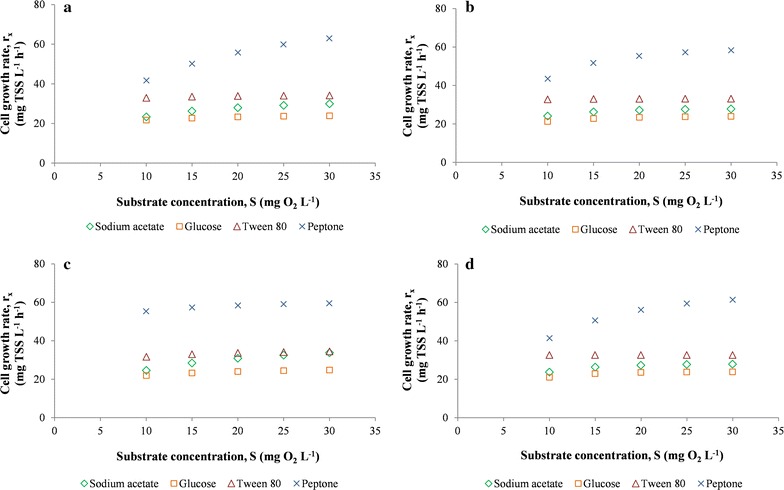
Cell growth rate (r_x_) of *Shewanella baltica* KB30 during the respirometric experiments in relation to substrate concentration (S), measured as chemical oxygen demand in (mg O_2_ L^−1^), obtained by different models: Monod (**a**), Moser (**b**), Contois (**c**) and Tessier (**d**)

The bacterial growth was also evaluated through r_x_ for the consumption of the various organic sources, as observed in Fig. 5. The highest r_x_ was obtained during degradation of peptone for all the kinetic models studied, as the values of Y_TSS_ were similar for the different substrates used. Therefore, the highest concentration of biomass produced by *S. baltica* KB30, measured as X_T_ depending on time, was recorded on peptone.

## Conclusions

This work represented the first attempt to characterize the kinetic performance of *S. baltica* during its growth on various carbon sources using several models. Actually, the use of different models, allowing best fitting for the consumption of each substrate, permitted a comprehensive approach to the question. The biodegradation of sodium acetate and tween 80 was best fitted to Monod’s model while the Contois’ showed the best adjustment of the experimental data for glucose and peptone utilization. As stated by the kinetic parameters, biodegradation of peptone was faster than that of the other carbon sources. Thus in *S. baltica* KB30, proteolytic metabolism appeared to be favored compared to lipidic and glucidic metabolism. The foregoing statements suggest that, in natural environments, *S. baltica* KB30 would contribute much more to the mineralization and recycling of protein and lipid than carbohydrates. In addition, its rather great competence in metabolizing lipids could find use in environmental issues (bioremediation), such as the removal of surfactants and/or emulsifiers. On the whole, the results obtained in this work contribute to fill a gap in the knowledge of this metabolically interesting cold-adapted microorganism, which deserves to be studied in view of possible applications. Studies in this sense are currently in course.

## Abbreviations

ANOVA: one-way analysis of variance
COD: chemical oxygen demand (mg O_2_ L^−1^)
DEB: Department of Ecological and Biological Sciences, University of Tuscia
DO: dissolved oxygen (mg O_2_ L^−1^)
K_C_: growth coefficient of the Contois function (mg O_2_ mg TSS^−1^)
K_S_: substrate half-saturation coefficient (mg O_2_ L^−1^)
LSE: least-squared error (h^−2^)
OC: oxygen consumption (mg O_2_ L^−1^)
OD_600_: optical density at 600 nm
PCA: plate count agar
R_S_: dynamic oxygen uptake rate (mg O_2_ L^−1^ h^−1^)
r_su_: substrate degradation rate (mg O_2_ L^−1^ h^−1^)
r_x_: cell growth rate (mg TSS L^−1^ h^−1^)
S: substrate concentration (mg O_2_ L^−1^)
*S. baltica*: *Shewanella baltica*
SD: standard deviation
*S. oneidensis*: *Shewanella oneidensis*
TSS: total suspended solids (mg TSS L^−1^)
X_T_: biomass concentration (mg TSS L^−1^)
Y_O2_: yield coefficient referred to oxygen
Y_TSS_: yield coefficient referred to the total suspended solids (mg TSS mg COD^−1^)
µ: specific growth rate (h^−1^)
µ_empirical_: empirical specific growth rate (h^−1^)
µ_m_: maximum specific growth rate (h^−1^)
µ_theoretical_: theoretical specific growth rate (h^−1^)
